# Southern Dental Association

**Published:** 1886-09

**Authors:** 


AMERICAN JOURNAL

OF

### DENTAL SCIENCE.

VOL. XX. THIRD SERIES—SEPTEMBER, 1886. No. 5.

ARTICLE I.

### SOUTHERN DENTAL ASSOCIATION.

### EIGHTEENTH ANNUAL MEETING.

### AT NASHVILLE, TENN., JULY 27, 28, 29, AND 30.

(Reported by Mrs. M. W. J.)

The eighteenth annual meeting of the Southern Dental Association was held in Nashville, Tenn., in Watkins' Institute.

The meeting was called to order on Tuesday, July 27, at 10:30 A. M., by the President, Dr. W. C. Wardlaw, Augusta, Ga.

The following officers were present:

President, Dr. W. C. Wardlaw, Augusta, Ga.

First Vice-President, Dr. B. H. Catching, Atlanta, Ga.

Second Vice-President, Dr. J. R. Knapp, New Orleans. La.

Corresponding Secretary, Dr. E. S. Chisholm, Tuscaloosa, Ala.

Recording Secretary, Dr. R. A. Holliday, Atlanta, Ga.

Treasurer, Dr. H. A. Lowrance, Athens, Ga.

EXECUTIVE COMMITTEE.

Dr. W. H. Morgan, Nashville, Tenn.

Dr. G. F. S. Wright, Columbia, S. C.

Dr. W. H. Richards, Knoxville, Tenn.

The meeting was opened with reading from the scriptures, and prayer by the Rev. I. D. Barbee, Nashville, Tenn.

The minutes of the last annual meeting, held in New Orleans, March, 1885, were read and confirmed.

Dr. Prewitt welcomed the association in behalf of the Tennessee Dental Association, and the local interests.

ADDRESS.

*Gentlemen of the Southern Dental Association :*

It has been made my pleasant duty to welcome you in the name of the Dental Association of the State of Tennessee.

First, I desire to acknowledge the honor conferred upon me, and my inability to fitly convey to you by word, the sincerity of that welcome. I am happy to know, however, that there are to you other evidences of your welcome than the words of the representative of the State Association.

While it has never been my pleasure, nor that of a number of gentlemen present, to meet with you in association capacity, yet you are no strangers to us. As a body of investigators, and as an educator, in so far as associations are educators, we regard you as second to none; though it may be less pretentious, possessing merit without assumption—seeking to spend and be spent in the upbuilding and advancement of that profession which, next to the wife and little ones, you love most.

Upon your rolls are the names of those whose reputaons are not confined to any section, but whose names are familiar to the profession in every clime. There are those here who were at the forming of this association, still active and aggressive; some who took passage when the ship was full rigged, and some who did but yesterday, as it were, enlist among you. Still, there are others, standing ready with credentials regularly made out, and waiting impatiently for permission to be numbered with you. To all we say, you are welcome.

True it is, that you have all, doubtless, expected an address of welcome, whether you were really welcome or not. In these piping times of peace, when all seems going as merry as a marriage bell, it is but a part of every programme—it is taken as a matter of course, that upon all occasions like the present, some gentleman will, with glittering speech and well-rounded sentences, after the fashion of form say: "You are welcome."

We would make you know, beforehand, if language were not so poor, what you can know only when you are gone; you come well, in that your coming promises not only a flow of soul, but a feast of reason.

You have lived, may be, for months, in the happy anticipation of this pleasant re-union, when you would shake hands with your brothers, and enjoy again that social intercourse which these meetings invariably develop. Nor is that all; you have been stowing away incidents of interest for this meeting, and have been plying with all your might the principles of science to every branch of the profession, that you might present matters of interest for the consideration of this body. That the strides made by the profession in the last quarter of a century are giant ones, is no argument that there are not yet many such to make; and to you the profession, as well as the people of your section, especially, are looking as a body of representative men, to perfect, as far as possible, methods, theory and practice.

I forget, however, that it is no part of my business to direct, or even forecast the deliberations of this body, but only to tell you, if I can, how welcome you are.

In that you are, in some manner, the guests of the Dental Association of the State of Tennessee, you are welcome. In that you come, a body of representative men of the profession, by dint of study, application and experience, prepared to discuss and demonstrate from every standpoint, matters of greatest interest to the dentist and his patient, you *come well.*

We rejoice, that while this word of greeting has been made to do duty under so many varied circumstances, yet on no occasion has it been intended to be more heartily employed. Except when used in *hollow form*, no word, perhaps, expresses more. None but the brave officer who cried in the agony of his soul when the fortunes of Waterloo were trembling in the balance: "Would to God night or Blucher would come!" can ever know how *welcome* either would have been.

When Cæsar had received from Brutus' arm, nerved by ingratitude, the fatal stab, he ceased to struggle, and, with the words: "Et tu mi filli," he wrapped himself in the more than royal robes of Rome, reeled, and fell at the foot of Pompey's statue, *welcoming death!*

Not till the Peri had brought a sigh of repentance was the gates of Heaven thrown wide open, and he *welcomed in.* Expressive, beautiful comprehensive, take it in its most expressive sense; and it is thus we mean it. Make the most beautiful application of it, and it is thus we would apply it. Make the broadest and most comprehensive use of it, and you have used it as we would.

While we welcome you, we know it is to work as well as pleasure, and it may be well for each to ask himself the question:

> "Why all this toil, for triumphs of an hour?
> Life's a short summer, man's a flower;
> By turns we catch the vital breath and die;
> The cradle and the grave, alas, so nigh!"

Why am I here? For what? Is it that this meeting may better prosper and fit me to serve my patients? Have

I in view the betterment of the condition of my fellow-man? Is it that by contact with the better element of the profession my views may be broadened, my capacities enlarged, my zeal increased? If so, then there should be no drones in this hive, but each one should contribute his mite, and he who feels himself not a good talker, or has nothing to say, let him see to it that he proves himself a good listener, and that he can watch as well as pray.

In conclusion, and in view of the very sacred and intimate relations existing between the dentist and his patient, and in view of the further fact that in the profession since this body last met, death has dealt his shafts both right and left, and whole battalions lie afield, it may not be out of place to admonish and remind one another to

> "Make then, while yet we may, your God your friend,
> Whom Christians worship, yet not comprehend.
> The trust that's given, guard, and to yourselves be just;
> For, live we how we may, *die we must.*"

Again we say, you are welcome.

Dr. G. H. Winkler, Augusta, Ga., responded in behalf of the Southern Dental Association.

He expressed his gratification at being made the mouthpiece of the Southern Dental Association; his appreciation of the kindly sentiments expressed, and of the general welcome tendered, which was only equalled by the gratification of accepting the same. He spoke of the giant strides made by Nashville; an advanced civilization, due to her men of energy, zeal, and capacity, whose hands had been upheld by women clothed in the purest of all womanly attributes. He spoke of her commerce, of her railroad facilities, her factories, founderies, and machine shops; her educational institutions; of her magnificent state house; of her numerous asylums; of her daily, weekly, and monthly publications, which are models of literature; of the spires and domes of her many churches, proclaiming a God-fearing people, concluding with the hope, that in justice to Nashville, every member of the organization would put forth his best energies, and all combine to make the eighteenth annual meeting the most memorable in the annals of the association; binding closer the ties of fraternal intercourse, inspired by good thoughts and noble deeds.

The President requested that the names of applicants be now handed to the Executive Committee, and voluntary essays to the Secretary. He tendered a welcome to the dentists of the city, to members of the medical fraternity, to the ladies present, with the hope that they would come again.

At the suggestion of Dr. E. S. Chisholm, the students of the various educational institutions were also included in the welcome tendered.

After a brief recess for the payment of dues, the society was called to order again, and Dr. B. H. Catching, Atlanta, Ga., in a few well chosen words, introduced the venerable Dr. W. W. H. Thackston, of Farmville, Va., the oldest living graduate in the profession; the only survivor of the second class of the Baltimore College of Dental Surgery.

In response, Dr. Thackston, as delegate to the association from the State Society of Virginia, spoke as follows:

DR. THACKSTON, OF FARMVILLE, VA.

Fourteen years ago, upon the banks of the "James," in a city setting upon her "seven hills," and looking mournfully down upon her battered walls and ramparts; upon her crumbling ruins, and upon the graves and monuments of her illustrious dead, it became my duty, as it was my honor and pleasure, to welcome you to all that was left of that grand old city, and to all that remained of that grander "Old Dominion," to her wasted homes, to her broken altars, to her humble hearth-stones and to her hospitable roof-trees; but amid all her wreck and ruin, to hands as clean, to hearts as warm, and love—love as pure as bannered knight, or crested cavalier ere dared maintain in "tented field," or glittering tourney.

The years have come and gone, and gone are some whose hands we then shook and clasped in the fond hope that we should meet again. Some who faithfully shared your every burden, who rejoiced in all your triumphs and who justly and worthily wore your proudest honors, and now and here, under the dispensation of a benignant Providence, I meet you again upon the banks of another beautiful river; in a young, a populous and flourishing city, instinct with life and energy, busy and prosperous, teeming with commerce and traffic, decked and crowned with marts of trade, with temples of art, with temples of science and temples of worship; and here in this honored and august presence, amid these grand and imposing surroundings—in this hall—I bring you once more the greetings, the salutations, the all-hail of that same "old mother" who still regarding you as, in a large degree, her own social, political and professional offspring, repeats the proud exclamation of the Roman matron when pointing to her children, "these—these are my jewels."

And, now, what shall I say in acknowledgment of the reception who have accorded me and my honored colleagues? What offering could I lay upon your altar worthy your acceptance and commensurate with your kindness?

It would be vain and presumptuous to essay the role of teacher, for you are adepts and experts in art, and proficients and scholars in science; you are not only my peers, but my masters. I bring you no new "dispensation," and can unfold for you no late "revelation;" I have solved no hidden problem, and can make no grand and imposing contribution to your stores of knowledge. Why then, you may ask, am I here? and I will answer: To meet you "face to face, to look into your hearts and to show you my own, to shake hands and touch elbows, to labor with and learn of you—for you can tell me what I never heard, and teach me what I never knew."

I am here with my honored colleagues as the joint bearer of the salutations and benizons of our "State Association;" here not only to greet you fraternally, but to tell you that we feel a pride in this federated organization, which is the legitimate outcome of the State Associations that Virginia was the first to establish and incorporate; to say that we recognize and honor you as the "upper House," as the Dental Senate of the South, and also to fling wide open our gates, our hearts and homes, and ask that you will once more honor the "Old Dominion" as your place of annual assemblage.

And in addition to this delegated and representative duty, there were personal considerations and attractions that irresistibly drew me hither. I desire once more to take counsel and compare notes with "old friends," and to meet and make friends of the young and rising representatives of our profession in the South and West; the men around whom cluster the fond and proud hopes of that bright and promising future to which our hearts and eyes are turned.

I desired to commune, and possibly for the last time on earth, with some of those who, in "days lang syne," trod with me the stony paths of our "wilderness," who tasted with me the "bitter waters" of our professional "march;" and notably of these: Dr. W. H. Morgan, the honored dean of Vanderbilt, who, with strong arm, with dauntless courage, and with abiding faith, for forty years has "smitten the rock," and who now beholds flowing around him the sparkling, crystal streams of a science no longer desecrated and repudiated, but recognized, respected, honored and taught in our "groves and academies," our colleges and universities, and more than that—*practiced* with a profit and beneficence to society that distinguishes no other era in our professional history. Thank God! this grand old man has been spared to see the seed he helped to plant in this virgin soil, germinate, grow and bud, and blossom, and at last bear fruitage to his labors, and we can only say may he "rest under the shade," and his declining years be sheltered, fanned and refreshed by the foliage he has so faithfully nurtured, and pruned and trained.

Another strong attraction, Mr. President, was your corps of Southern and Western editors and journalists—the gentlemen, not of our "*fourth*, but eminently of our *first* estate," the gentlemen who spread our monthly and quarterly feasts and banquets, who trim our sails, who stand at the helm and navigate our barques; they who dress our thoughts and clothe our ideas, and shed upon our pathway the meridian light of this wonderful day of progress and professional development.

For these reasons and considerations I have left my home, have deferred pressing duties and obligations to accompany my fellow delegates, and be with you to-day. And, now, I will only trespass upon your time and attention to again thank, and tell you that I shall cherish this occasion and the reception you have accorded me, as one of the ever bright and green spots in life's long journey; as the fulfillment and realization of my highest aspirations and as a princely recognition of my claims to your consideration.

And, now, in closing this reply to the knightly, courteous and fraternal introduction I have received, I shall presume upon your kindness, and beg an humble place in your ranks while we are together, and a small niche in your breasts when my life's work is over, and I have become to you—and to all—a memory and a shadow.

The President, Dr. W. C. Wardlaw, delivered his annual address.

PRESIDENTIAL ADDRESS.

*Gentlemen Southern Dental Association:*

In discharging this constitutional duty which your kindness has devolved upon me, I desire to address you a few thoughts upon the relationship of dentistry to the science of medicine.

Is dentistry a distinct profession, or should it be regarded as a specialty of medicine? In royal old England, so conservative of customs and so jealous of dignity, the latter claim would not be allowed, but in democratic America, a more liberal sentiment obtains, and such a relation is gradually being accorded.

In England to-day, the College of Physicians, those mighty men of powdered wig and gold-headed cane, frown down "specialists," even in surgery, and the business of the apothecary, and their conservative instincts still protest against "specialism." The battle is even now raging there, with victory smiling on "specialism." As it is conceded that America has been the leader of England in most matters pertaining to dentistry. we may hope that in this instance, too, she may succeed in breaking down some of these conventional barriers standing in the way of progress.

Our claim is, that whilst dentistry is a distinctly organized profession, made so by peculiar circumstances, it is, properly and really, a "specialty" of medicine, being, at the same time, a science as well as an art, and should be so recognized and encouraged. The medical fraternity generally are disposed to regard it as only one of the arts, with a slight admixture of medicine.

Medicine, in the present acceptation of the term, is very comprehensive, and signifies all that pertains to the prevention, alleviation and cure of disease, being conveniently divided into surgery and medicine. The ancients did not recognize a distinction between these two, but included both in the "healing art." We, however, mark the essential points of difference, by limiting surgery to the concern of local injuries and disorders, and restricting medicine to those affections which involve the general system. Homer does, in his "Siege of Troy," allude to the two sons of Æsculapius, as being, the one a skillful surgeon, and the other a wise physician, but that this separation was very marked or long-continued, history does not inform us.

General surgery is arbitrarily subdivided, according to the particular part or organ treated, into opthalmic surgery, obstetric surgery, laryngeal surgery, oral surgery, etc. Dentistry, being almost synonymous with oral surgery, would thus seem to be a part, and a special part of medicine. But not having been so taught by medical colleges, and rather repudiated by them, how has it been evolved as a "specialty" of medicine?

The vast and ever increasing domain of medicine would seem to preclude the possibility of the whole of it being properly cultivated by one mind. Necessity and convenience have, therefore, divided and subdivided it into various branches and departments. Long before the accruing knowledge of modern times embraced this necessity, the ancients had found it expedient to do the same thing. Herodotus tells us that in Egypt, "the mother of the arts and sciences," so wisely was medicine managed, there were physicians, "each of whom applied himself to one disease and only one; some are for the eyes, others for the head, others for the teeth, and others for internal diseases." This system, however, did not prevail generally, and during the slow advance of many years, the individual practitioner was expected to possess all attainable knowledge of the healing art.

But, as each upward step upon the mountain side widens the range and extends the view, successively bringing to sight new and varied beauties of nature, until the farthest reach of vision is unable to take it all in, so the onward march of late study and research in medicine has lead to the discovery of new truths and the development of new principles, until now no one intellect is of such giant proportions as to be able to grasp them all. Necessity, I say, has therefore decreed that it should be parcelled out amongst a number of co-laborers, each pursuing that branch recommending itself to his preference, and so, one and another becoming skillful and proficient in his special branch, would naturally be acknowledged as an "expert," the modern "specialist." This was, perhaps, first observed in the disposition of some physicians to limit their practice to that of surgery proper.

Division of labor, mental and manual, tends to proficiency of attainment. We may ridicule the man with a "hobbv" as the one-idead man, but it takes the man with one idea, persistently and energetically following it, to carry it to a successful issue. A jack-at-all-trades is very often good at none. He is most apt to excel in art or science, who, in the choice of a life vocation, is wise enough to select that one most congenial to his taste and in accordance with the bent of his genius. Having so chosen, and through ambition and application made a success, it is his mission to pursue it, because therein he can do the most good for his fellow-man.

The modern " specialist " is, therefore, a deservable and legitimate personage. Accordingly, we have in medicine, the specialist of the eye, the ear, the thoracic cavity, the urinary organs, of nervous disorders, of skin diseases, uterine affections, etc. And why not the specialist of the mouth?

This condition of affairs, however, has only been brought about after a long and bitter contest. Only recently in America, the great Marion Sims was bold enough in courage and sufficiently independent in fortune and reputation, to break away from the trammels of arbitrary custom, and successfully advocate the professional recognition of " specialists." In comparatively late years, it was against the " code of ethics " of the profession—'twas quackery——'twas undignified, unbecoming, for a regular practitioner to claim, however modestly, special merit or superior skill in any particular department to which he may have given attentive study.

This prejudice still obtains to a certain extent, and seems to attach especially to dentistry. Why, I am at a loss to understand, unless it be that in its practice it has much of art, and the mechanical arts do not recommend themselves to that pride of intellect which is accustomed to look with contempt upon manual labor as not altogether respectable. General surgery is art, high art—utilizing numerous mechanical instruments and appliances, and requiring dextrous skill, an educated eye and a just perception of the beautiful. These are essential elements of dentistry, and it should not be accorded any less respect and honor on account thereof.

The cases are similar thus far, but the difference which seems to make the distinction, is, that dentistry has not been acquired in a medical school as surgery has been. Dentistry, as a matter of fact, is not covered by a medical diploma, and to that extent cannot technically be a "specialty" of medicine. What is a diploma, however, but a mere certificate of proficiency? If bestowed upon one lacking the requisite qualifications, it no more makes him an M. D., than does the withholding of it from a worthy man make him any the less a doctor in reality.

Dentistry, from the very nature of things, has grown up outside of medicine. 'Tis like the deserted child who, dis-owned by its mother, and brought up on the bottle, survives in spite of its hard usage. Medicine had already stepped forth in the pride of well developed proportions when dentistry began to take its infantile steps in its practice as a rude art by the uneducated barber. But, as one by one, men of observation would follow out its practical teachings to their logical results, 'twas found that the fundamental principles of medicine underlay them all. These convictions gradually enforced themselves, until the ambitious artist felt impelled to closely study those branches of medicine which he recognized as bearing most intimately upon his own calling. He studied medicine in dentistry because he could not study dentistry in medicine. The wants of her unrecognized child had not been foreseen and provided for by the unnatural mother, and only as circumstances allowed, could they be supplied by itself.

The upward progress was toilsome and tedious. Dark ignorance had to be enlightened, contracted selfishness had to be overcome, blind prejudice had to be dispelled, the sunlight of truth and science had to be admitted. All of this had to be accomplished without the encouragement and against the opposition of the medical fraternity. Pride, prejudice and jealousy took the place of intelligence, penetration and courtesy. Our reverend friend, Dr. Thackston, remembers of his own personal knowledge and experience, how Dr. Chapin A. Harris and his coadjutors made overtures to the medical colleges, endeavoring to have dental professorships incorporated in them; how their propositions were scornfully rejected; how they were driven to the necessity of establishing an independent school; and how the Baltimore Dental College, the first dental college ever founded, had to struggle and strive for its early existence. And thus it is. Dentistry came to be taught in separate schools and to be independent of medicine. The dental schools, however, are mere duplicates to the medical schools in anatomy, physiology, pathology, chemistry, materia medica, etc., and the dental student might study these branches as well in one as the other. As time and research extends the spheres of both professions, the necessary provisions will probably be made for M. D's., and dentists being taught and graduated by the same corps of teachers. Harvard University has the honor of having led in this direction, and has been, and will be, followed by other universities. My own judgment leads me to think that dentistry pure, will be better taught in dental colleges.

Year by year, as our standard of qualification is being raised, men of character and education are joining our ranks and are being recognized by physicians as professional equals, being called into consultation with them as occasion may require.

Let us each, therefore, feel resting upon himself individually, the responsibility to uphold the honor and maintain the dignity of our loved profession, and there will be no need of cringing and fawning to secure that honorable recognition of which we are worthy.

It was my good fortune recently to attend a meeting of a State Medical Association, and I was surprised and mortified to learn the very low average grade of education of medical students generally, and the comparative ease with which a medical diploma may be obtained.

Their State laws are very imperfect, affording no reciprocal protection. They have no examining boards, and nothing like an Association of Faculties. Any bogus diploma will pass muster, and any ignoramus or brass-cheeked pretender may legitimately go forth to practice destruction. In the great State of Georgia there seems to be no legal method to arrest the depredations of impostors and mountebanks. At the very time the Georgia State Dental Society was successfully prosecuting, in the city court of Augusta, violators of the law against the illegal practice of dentistry, a committee of physicians was running around consulting lawyers, hunting offices, and devising measures, in the futile effort to prevent an oily-tongued, jewel-bedecked and brass-mounted quack doctor, from maltreating the citizens, and reaping a rich harvest of ducats, from the hard earnings of his deluded victims. They were really powerless to do more than grit their teeth and look on with smothered curses.

But I merely touch on these educational matters, knowing that we are to enjoy a rich intellectual repast, in some of the papers to be offered on them. Other matters looking to our higher advancement, and suggesting them selves for consideration and action, might be mentioned: the appointment of dentists to the Army and Navy, and on the National Board of Health, our share in Congressional appropriations for scientific research, the approaching International Medical Congress, &c. But I have done.

Gentlemen, has not Dentistry, with her many inventions, valuable discoveries, and signal triumphs, fairly earned for herself a niche amongst the sciences, and has she not before her a future of glorious possibilities?

Only two Sundays ago, I heard for the first time in my life, specific and honorable mention made in a public address, of dentistry as a distinct profession, with no allusion to, or connection with medicine. An eloquent minister of the gospel, in glowing and complimentary terms, referred to it as a new science, which had taken its place among the highest and most important professions of the age.

I thank you for your courteous attention.

Dr. W. H. Morgan, chairman of the Committee on Arrangements, announced that daily sessions would be held from 9:30 to 12:30, and 2:30 to 5:30. Friday being set apart for clinics.

The association then proceeded to the regular order of business.

REPORT OF THE COMMITTEE ON DENTAL ASSOCIATION.

*Committee :* Drs. B. H. Catching, Atlanta, Ga.; M. C. Marshall, Little Rock, Ark.; G. W. McElhaney, Columbus, Ga.; J. B. Patrick, Charleston, S. C.; W. D. Dunlap, Selma, Ala.; F. J. S. Gorgas, Baltimore, Md.

Dr. B. H. Catching, chairman of the committee, read a paper entitled, "Medical Education for Dentists and Dental Education for Physicians." (Will appear later.)

Other papers on the same subject were deferred to the next session.

The Executive Committee recommended the following applicants for membership:

Drs. J. Crawford, Nashville, Tenn.; G. Chisholm, Columbus, Miss.; G. S. Staples, Sherman, Texas; J. M. Lunquest, Birmingham, Ala.; L. D. Wright, Dixon Station, Tenn.; H. W. Morgan, Nashville, Tenn.; W. D. Taylor, Brownsville, Tenn.; H. H. Barr, Oxford, Ala.; J. H. Prewitt, Madisonville, Ky.; G. Eubank, Birmingham, Ala.; J. S. Franklin, Nashville, Tenn.

On motion, the rules were suspended, and no objections being made, the Secretary was instructed to cast the ballot.

The election being unanimous, the newly elected members were invited to sign the Constitution and pay their dues.

On motion, adjourned to 2:30 P. M.

---

TUESDAY, July 27.

FIRST DAY—SECOND SESSION.

Called to order at 3 P. M., the President in the chair.

The Executive Committee announced the following applicants for membership:

Drs. D. R. Stubblefield, Nashville, Tenn.; R. B. Adair, Gainesville, Ga.; I. B. McDonald, Shelbyville, Tenn., who were duly elected.

DENTAL EDUCATION.

A second paper bearing this title, by Dr. W. D. Dunlap, of Selma, Ala., was read by Dr. B. H. Teague, of Aiken, S. C. Also a third, by Dr. M. C. Marshall, of Little Rock, Ark.

The subject was then declared open for discussion.

Dr. J. J. R. Patrick, of Belleville, Ill., said: This is a stupendous subject. It is like the harp of a thousand strings: the more you play with it the more music you get out of it. In every society in the United States this question constantly arises. The point most universally, but in my opinion most unnecessarily agitated, is whether or not dentistry is a specialty of medicine. Any attempt to answer this constantly recurring question necessitates a retrospective view, for our profession, like everything else in this wide world, has its history.

If we make medicine the standpoint, it is necessary to know upon what it stands; what the degree of M. D. is supposed to represent. It represents a knowledge of physiology, anatomy, pathology, chemistry, botany, geology—all the sciences; that's what medicine represents. The wisest man of his tribe was the "medicine man."

It is not so very long since the time when any man who devoted himself to any one branch of the healing art was considered a quack by the general practitioner. If he treated only the ear, or the eye, or the teeth, he was a quack; no matter what his education, his skill, his talents, he was a backslider of a specialist. Now there are many schools of medicine—the allopathic, the homœpathic, the electic, the electric, and all give the same degree of M. D. It is not a question of schools; they are all willing, all proud, to become the godfather or godmother of dentistry.

What was medicine up to the time of John Hunter? From the time of the Greek and the Roman empires not one step in advance was made. Medical works, up to the fourteenth century, were all compilations from Greek and Roman authorities. Esculapius claimed to have invented a forcep for the extraction of a tooth, but the ancients knew little or nothing of anatomy or physiology.

Thomas Knox, of Edinburg, was the first anatomist, and that is as late as 1828. The anatomist was anathematized. No believer in a literal resurrection could santion the cutting up of the body of a human being.

John Hunter was the first one who described the fœtal circulation; nothing was known of the diseases of women and children before John Hunter; no one had even described the human jaw, the human teeth. Physiology was nothing at all, until John Hunter made it; it was only a wild theory. And yet he was not a surgeon; he was a clumsy operator. He was a comparative anatomist, but he was not an able man. He was but a wild young man when he went into his brother's office. He taught us that the different preparations of mercury would destroy insect life. [Time up.]

Dr. Wm. H. Morgan: Much that has been said I can indorse most heartily. Dentistry undoubtedly has a history, but some erroneous statements have been made. It was in 1836 (and not in 1840 as stated in one of the papers read) that the first dental association was formed, and it was not the American Dental Association as stated, but the old American Society Dental Surgeons. Up to that time there were no sources for a dental education. When I began to study dentistry, the good old gentleman who undertook my education, told me there were but two authors on the subject: Bell and Harris. The old *American Journal and Library of Dental Science*, was the first fruits of that society. Realizing the inefficiency of office-teaching, that society soon began to discuss the propriety of establishing a school where dentistry could be properly taught. But very few dentists would receive dental students. One offered to qualify them for $300 in the city, or from $600 to $900 in the country. The first idea conceived was of a dental school, in connection with a medical college, to make it respectable. But the dental students were treated with so much contempt that the idea was abandoned, and an independent dental college organized. Dentistry took care of herself then, and I have a suspicion that she will be able to take care of herself in the future. The college was at first but a small affair, with only but four chairs—one of anatomy and physiology, one of the institutes of dentistry, one of mechanical and operative dentistry, and a fourth which took in a little of therapeutics, a little of materia medica, and a little of everything else.

This college—the Baltimore College of Dental Surgery—has so grown within seventy years that physicians may be found studying anatomy and chemistry in her halls, because they find better facilities there than in the great universities.

And yet it was said in a paper just read that their scope is not broad enough!

Again, it was said that English methods were better than ours! Why? It is well known that the good dentists, few and far between, in Great Britain are all American born, educated here; and so it is in France, in Germany, in Russia. When Gov. Brown was minister plenipotentiary to Russia, the British minister sent to him to ask where he could find an American dentist for his family? American dentistry leads the world, because it is based on a system of thorough college education, which is the outgrowth of society and association work. It is true that our associations are in themselves educators, but if a man had to depend on them alone for his dental education, he would stop short.

As to a medical education, there are branches taught in the medical college which are not taught in the dental college; the place to make a medical man is the medical college; the place to make the dentist is the dental college. [Time up.]

Dr. Catching, of Atlanta, Ga., said: No one disputes the progress made by dentistry in a practical way. It is true that American practical dentistry leads the world. But a man cannot get that medical education from a dental college which will entitle him to recognition as practicing dentistry as a specialty of medicine.

Dr. Morgan was, probably, a remarkably bright youth and he has continued to grow brighter to this hour. But, when Dr. Morgan married and a son was born to him, who, probably inherited the talents of his father, he was not satisfied to qualify that son for the practice of dentistry, in a dental college. He wished to give him all possible advantages, and he was educated at a medical college, and graduated as an M. D., and he stands to-day an M. D., and also D. D. S. That tells the whole story.

Formerly, large numbers of European students sought their dental education in our schools, but now Europe is independent of America. Germany almost denies admission to American dentists; they may practice, but they must not teach.

Dr. Morgan: Is not Dr. Miller, to-day, the most prominent, the leading Professor in the University of Berlin.

Dr. Catching: She does not propose to turn out those who are already holding positions there. In looking at the matter from a college standpoint, it seems to me that Dr. Morgan is speaking for the shekels——

Dr. Morgan: I resent that as a slander. No man can impugn my motives——

(After some excited simultaneous remarks on the part of Drs. Morgan, Catching, Salomon, of New Orleans, and others, order was restored, and Dr. Catching continued:)

Dr. Catching: A college Professor cannot look at this matter in an impartial manner; he looks at it from a college standpoint; from the windows of Vanderbilt college; from the standpoint of the Dean of a Department. I speak simply as a member of the profession, and I must be allowed to speak plainly. A graduate of Vanderbilt told me, not three months ago, that he considered that when a dentist reached up into the antrum of Highmore, he was getting out of his legitimate field! We cannot stand still; we must either go backward or go forward. The world, and professional pride, demands that we go—not backward, towards the barber-shop again, but forwards, into the science of medicine. The practical field is large, but if we wish to attain the highest standard, it must be through a medical education. To graduate only as D.-D. S., has ceased to be a benefit; it acts as an injury to our advancement. We should enter a reputable medical college, and come out as M. D., and then enter a dental infirmary, where the practical work of dentistry is taught by the very best men. When we come out from the medical college and the dental infirmary, we shall be entitled to recognition as practitioners of the dental specialty of medicine.

Dr. Salomon, New Orleans, said that Dr. Catching had portrayed the ideal future of dentistry, but not what was possible in the present; that coming from Germany, yet what he knew of dentistry, he owed to the Baltimore College; but that things had changed for the worse since 1870. New American colleges had been established, which take men in who can scarcely write or read; who are only fit to stand behind the plough or the work-bench. Germany now stands the highest, for the dental school in the University of Berlin registers a very high standard of education. How many dentists understand the effects of uterine affections, or of menstruation upon the teeth? Either dental schools must afford broader medical education, or medical schools must enlarge their dental facilities. American graduates ara not allowed to use the title, "doctor," in Berlin, but must graduate again. One thing that has degraded American dentistry in Europe is the fact that ignorant German office-boys, who have saved two or three hundred dollars from their wages, and picked up a few stray technical phrases; who have "studied" for many years while sweeping out offices and washing spittoons, come and spend eight or ten months in America, and go back as Doctor So-and-So. It would be a shame and an outrage to cast reflections on our Alma Mater, but it is our duty to urge our colleges to enlarge their sphere of usefulness ; not casting reflections on the old colleges, nor seeking to abolish them, but looking to higher standards for the future.

Dr. Morgan: Allow me one word in reply.

President Wardlaw: When all who desire to do so, have spoken once.

Dr. B. H. Teague, Aiken, S. C.: We all know that a great deal of good comes out of these discussions. Much benefit may be gathered from the remarks of those who have spoken. Dr. Morgan was timid, so to speak, in expressing himself, knowing that he occupied a position easily assailed, being on the dental faculty; but we know him to be a pure, upright man, and a good educator. Dr. Catching is equally pure and upright, and equally interested in the question of dental education ; but each one looks at it from a different light; it is only necessary that they should understand each other. What Dr. Salomon has said is true in every respect, for it cannot be denied, that of late years American dentistry abroad has brought disrespect upon the profession. Germany is of a jealous nature; she will not, knowingly, admit an enemy within her borders. Her public has been imposed upon by our graduates, and our American colleges require overhauling. We graduate men who are not educated men. The trouble lies at the threshold. A young man who holds a pseudo certificate that he is "a good student," is not asked whether he can write a good hand; whether he has graduated from a literary college, or from a good high school! Often he is allowed to use the name of some unknown member of the profession as preceptor; uses the infirmary a few months in summer, and not having much money to spend, graduates at the end of the second term; but that diploma does not make him a dentist. But I believe in dental colleges. I am glad I am a dental graduate, and am only sorry that I am not a medical graduate as well. A man cannot get a diploma from a literary college if he is not an educated man. President Garfield had to work a long time to pay his way through. If the qualifications for admission to the dental colleges were of the proper standard, we should have fewer unwon diplomas. Medical colleges experience the same difficulties. We should require the student to be an educated man, and we should see that his preceptor also is an educated man.

Dr. H. J. McKellops, St. Louis: This is a very broad question. I do not stand before you as a graduate, myself: I am a self-made man. I earned the money for what little education I had, and there are plenty of such men. We all see the want of education. When I look around upon these young men, and see the opportunities they enjoy, I cannot but exclaim: "Oh God! if I only had the chance they throw away so lightly!" I paid dollar for dollar for all I ever got. If it was a receipt for soft solder, I had to pay for it, and I had to make the money myself. I love my profession, and I love every man it. I put the latch-key out, and say to every man: "You are welcome to my office, and to learn all you can in it." My patients never refuse to let my professional brethren stand by my chair. I never say to them, as has been said to me: "Excuse me, but Mrs. So-and-So is in the chair, and she does so hate to be seen!" I train my patients differently. Last May there were 2,200 doctors in St. Louis, and they did not turn their back on dentistry. Many of them are members of both professions; they are the favorites of one and the pride of the other. But dentistry is bound to be a separate profession; it is not part and parcel of medicine. John Hunter was no graduate; he was a poor, miserable boy before he got into the office of his brother. I wish every young man would study the life of John Hunter. He would see that it all lies with himself. Only his own personal exertions can place him on the top round of the ladder. If you would make your mark, it lies only with yourself. A few years ago, I saw six or seven German women, who could not speak a word of English, studying dentistry in a college at Philadelphia. Graduates from the Michigan college, or from Harvard, can go to London and practice without an examination. In Paris and Germany, they are waking up. I went to New York to see Mr. Herbst operate. I stood by him ten days. He has no patents to sell. He freely gives all he has. He says: "I have brought my baby to this country—I want to show it to you. If you don't like it, I'll drown it." That man has genius. As to Professors in our colleges—there is no money in it. It is time lost and money out of pocket.

In response to urgent calls, and at the request of the President,

Dr. W. W. H. Thackston, of Farmville, Va., took the floor. He said that in the earlier efforts toward dental education, alliance was sought with medical colleges and medical departments of universities, but we had been driven to rely upon our own resources; and without egotism or boasting, he could now feel just pride in the condition of dental surgery, of dental science. For centuries medicine had entire control of the field. Harris, Hayden, and the Parmlee's were the fathers of American dentistry. They had instituted an independent system of education, and had a right to be proud of its results—in the men it has made—and now this system is sought to be abrogated—why? Because some men under it are not what they ought to be! American dentistry leads the world, and the American system of education is the best in the world; but no system whatever can force ideas through thick skulls, or fill empty heads with brains. Other professions have their *quacks*, as well as ours; we do not have all the noodle-heads. There are some in medicine, and some in law, and some in the ministry. This independent system ought not to be hastily abandoned, though it may be improved. It would be making too great concessions of dignity and self-respect to go back now to the medical schools which so long gave us the cold shoulder. We can raise up a class of men to do us honor without going to the medical schools. One point that has been overlooked is, that there are few men who are fit to be dental cadets. Men who might make a distinguished reputation as physicians, or as lawyers, are not always fit for dentists. Medical students never have done, and there is no reason to believe they ever would do, better than dental students.

When the millenium comes it will make very little difference whether we attend medical or dental schools, but until that time let us be careful not to make any serious departures from established practices.

Dr. G. F. S. Wright, Columbia, S. C.: The subject is one of great interest, and on this, as on many others, we require frequently to be reminded of what we know. State Dental Associations have been the leading influence to foster training in dental schools, and during the past twenty years laws have been enacted in many of the States to regulate the practice of dentistry. It is a principle in common law that the law cannot make a crime of what was not a crime before the law was made. Men cannot go into a State where there is an efficient dental law, and practice dentistry unless qualified, but men already in practice cannot be interfered with. They have to be accepted with all their defects; we cannot make them go to college and acquire what they lack. The gentlemen who are so enthusiastic lose sight of what is being done in this direction. It will not be long before every State in the Union will have a law as efficient as that of Georgia, where a man cannot practice unless he is really qualified.

Dr. Jas. Johnson, Staunton, Va.: I love my profession. Surgery was my first love, but I left it for dentistry. I am glad to learn there is one State where the law has proved effective. In Virginia we have just passed such a law; before we could not get what we wanted, but now we have the law, and hope we can make it effectual. It is true, there are a great many now in practice whom we cannot reach; we cannot educate them. We shall have to be patient till they die off, for we cannot touch them. As things now stand, we need some change. I have looked the matter over carefully and think what we need is the establishment of universities with endowments. With well endowed chairs, we could get the best that the profession has. But as things are now we cannot obtain the best talent for our colleges; we have not the means to pay for it. The University of Michigan, with its endowment of $10,000,000, has grand opportunities. This is also true of Baltimore and Harvard.

Dr. Morgan: Baltimore is not endowed.

Dr. Crawford, Nashville: Dentistry draws from all other callings. It is the rarest thing in the world for a dentist to leave his profession and go into other business; but men from all occupations come to us. There is a fascination about it even in its present crude state. In a large per cent. of the literature of dentistry, fundamental principles are viewed from a medical stand-point. In our periodical literature the best articles come from men who sign M. D., with perhaps the addition of D. D. S., but the M. D. takes precedence.

The idea that "the dentist must be a doctor," has come to stay. The man who has the training and education necessary to receive that M. D., comes to the front. One idea which has been much talked about, ought to be repudiated—viz: that because Dr. So-and-So graduated at such an institution and is not competent, therefore that institution is to be condemned. We should not discourage our own institutions. They have turned out many good men, even if there have been some failures. We should extend a helping hand; help to correct their errors, but not abuse them.

I repeat, however, that the idea that a dentist must be a doctor has come to stay. [Applause.]

Dr. Morrison, of Nashville, said that he got his degree from Baltimore; that he had earned the money for his education between the plough-handles. He did not want the degree of M. D. A Latin scholar, five years away from his books, could rarely translate ten lines. Had seen diseased antrum of Highmore successfully treated by a man who never went to college; without instruction or text-books, by intuition almost, he was successful. He had also heard another, who boasted of his attainments, talk of "cutting out an ingrowing toe nail clear to the cementum!"

Dr. Stubblefield said that he occupied the same vulnerable position, as he was connected with a dental college and might be critized. Dental education was no longer a mooted question. It was uncalled for to talk of *shekels*. A man who expected satisfaction would go to an expert in any line. If it was a question of metals, he would go to a metallurgist; if a question of law, he would go to a lawyer; if on dental education, he would go to an educated dentist. If we could get every dental student to first graduate from a literary college, or even a high school, we would have a broad foundation on which to put the cap-stone. But we have few graduate-students, and must do the best we can with the material that comes to us.

We all honor Dr. McKellops. If, with the fine material which nature gave him, he had also had the educational facilities of to-day, what might he not have been? Native ability, thoroughly educated, can accomplish everything. Those who cannot hold out in the battle of life, have not the nerve to stand and fight it out.

I left the medical profession to be a dentist, and have never regretted it. God gave me mechanical instincts which find their proper outlet in dentistry. Special dental schools have their merits and demerits, but whether educated in a dental college, or in a dental department attached to some other school, makes but little difference if the man be of the proper temperament. "A man's a man for a'that."

Education means "leading out"—the better educated a man is the more capable he is of exercising his mental faculties; he is better developed, with power for better work.

Dr. Morgan rose to a question of privilege. He said I have made it the rule of my life to eschew personalities, and to conduct myself properly before my brothers. But I cannot permit any one to impugn my motives, or to insinuate that I am capable of dishonest work for the sake of money. I desire to tender my apologies for the hasty language I used, but I will say that I have spent $100 for the advancement of the profession where I have received five. My own education was very limited. If the rules then had been as stringent as they are now, I should have been shut out. I knew how to read, write and cipher, but I have worked on the streets of this city for forty cents a day since I became a man grown.

There has been a constant intimation here to-day that when men have attained a certain degree of education they are *entitled* to a degree; that the man who has studied and understands medicine is a doctor. This is all a mistake. Doctor is a degree conferred, on certain conditions, and a man cannot be a doctor unless the degree is so conferred, and it is practically a fraud to call a man doctor upon whom such a degree has not been conferred. A man may be a physician or a dentist, but he is not a doctor. The degree is conferred on certain conditions, and unless those conditions are complied with, not all the fame or the knowledge of an archangel *entitles* him to it.

Dr. McKellops: If a man presents himself for examination and passes honorably, is he not entitled to a diploma?

Dr. Morgan: No, sir; the institution requires that certain conditions shall be complied with, certain fees shall be paid, a certain curriculum passed, and a man is not entitled to a diploma until he has complied with all these conditions. The intimation has been made that I am not in favor of a broad education. Why: I have fought for that very thing for years. Every professor in the institution with which I am connected has M. D., and I had something to do with their selection. I chose them because I thought they had the requisite qualifications.

Some men have queer ideas of science. Something was said about young men who were taken into an office until they could polish plates, and on a certificate to that effect, enter college. I tell you if a young man can make a first-class polish on a gold plate he knows the science of it. He has done it, and he knows how it is done.

The proportion of thoroughly educated men in medicine and in dentistry is largely in favor of dentistry. I may say ten to one, in proportion to the number engaged in the two professions. It is not what a man knows when he goes into the college, that makes him what he is, but what he knows when he comes out.

As a rule, any office-teaching we get to-day from the average dentist, is a disadvantage. I would rather take a young man from behind the plough. It is a mistake to think that office training fits a young man for the dental college; it is wrong to the very core. Neither do I see the necessity of teaching all the branches of medical science, to make a man a practical dentist. What use has he for midwifery or pharmacy? of how to treat yellow fever or cholera? When he comes to practice dentistry he will have no use for it. If there is a case of cholera in the neighborhood, he will want to get away from it. Let him prepare himself for dentistry, on a broad foundation, but lay aside all collateral branches. The respectability or the disgrace of dentistry rests on your shoulders.—*Southern Dental Journal*

